# Prevalence and Genetic Diversity of *Legionella* spp. in Hotel Water-Supply Systems in Latvia

**DOI:** 10.3390/microorganisms11030596

**Published:** 2023-02-27

**Authors:** Olga Valciņa, Daina Pūle, Juris Ķibilds, Andžela Lazdāne, Jūlija Trofimova, Svetlana Makarova, Genadijs Konvisers, Laima Ķimse, Angelika Krūmiņa, Aivars Bērziņš

**Affiliations:** 1 Institute of Food Safety, Animal Health and Environment “BIOR”, 1076 Rīga, Latvia; 2 Department of Water Engineering and Technology, Riga Technical University, 1048 Rīga, Latvia; 3 Department of Metabolic Genetics Laboratory, Children’s Clinical University Hospital, 1004 Rīga, Latvia; 4 National Reference Laboratory, Riga East University Hospital, 1038 Rīga, Latvia; 5 Department of Infectology, Riga Stradiņš University, 1007 Rīga, Latvia

**Keywords:** *Legionella*, water, hotel, sequence type

## Abstract

*Legionella* is one of the most important waterborne pathogens that can lead to both outbreaks and sporadic cases. The majority of travel-associated Legionnaires’ disease (TALD) cases are contracted during hotel stays. The aim of this study was to evaluate the prevalence and genetic diversity of *Legionella* spp. in hotel water supply systems in Latvia. In total, 834 hot water samples were collected from the water systems of 80 hotels in Latvia. At least one *Legionella* spp. positive sample was detected in 47 out of 80 hotels (58.8%). Overall, 235 out of 834 samples (28.2%) were *Legionella* spp. positive. The average hot water temperature in Latvian hotels was 49.8 °C. The most predominant *L. pneumophila* serogroup (SG) was SG3 which was found in 113 (49.8%) positive samples from 27 hotels. For 79 sequenced *L. pneumophila* isolates, 21 different sequence types (ST) were obtained, including 3 new types—ST2582, ST2579, and ST2580. High *Legionella* contamination and high genetic diversity were found in the hotel water supply systems in Latvia, which, together with the insufficient hot water temperature, may indicate that the lack of regulation and control measures may promote the proliferation of *Legionella*.

## 1. Introduction

Bacteria of the genus *Legionella* are Gram-negative, non-spore-forming, rod-shaped, aerobic bacteria. Currently, more than 60 *Legionella* species are known, but more than 90% of Legionnaires’ disease (LD) cases are caused by *Legionella pneumophila*. *Legionellae* are ubiquitous, they have been found in underground and surface water, and wet soils, but their main reservoir is man-made aquatic environments, especially hot water systems in residential buildings, hospitals, nursing homes, hotels, and other private and public buildings, where they can colonize taps, shower heads, cooling towers, spas, and fountains [1,2]. Very low concentrations of Legionella in natural habitats can increase markedly in engineered hot water systems where water temperatures are below 55 °C [3]. Human infection with *Legionella* spp. is known to result from the inhalation of aerosols containing infectious bacteria [4]. 

Legionnaires’ disease caused by *Legionella* spp., manifested as severe pneumonia with a fatality rate of up to 15% and Pontiac fever—a mild, self-limiting flu-like disease [5]—belongs to sapronoses, opportunistic infections caused by free-living organisms that can under some circumstances multiply within a host [6,7]. Humans are not the principal target of these bacteria, as *Legionellae* can infect a wide variety of amoebae and protozoa and have evolved specific mechanisms to avoid the digestive system of free-living protozoa. The same virulence factors that help *Legionella* to infect protozoa also facilitate the infection of alveolar macrophages in human lungs [8]. Free-living protozoa provide *Legionella* with nutrients and additional protection from environmental conditions such as temperature fluctuations and disinfectants [9].

A balanced and multidisciplinary approach to prevention can be essential for reducing the incidence of Legionnaires’ disease; however, no mandatory preventive actions or regulatory norms with regard to hot water temperature have been defined for hotels in Latvia. 

Most cases of Legionnaires’ disease are sporadic and community-acquired, but some are associated with travel and medical facilities (travel-associated LD, TALD). Although an increase in the number of TALD cases has been reported up to the year 2018 across Europe, 67% fewer cases were reported in the EU in 2020 than during the previous year due to the COVID-19 pandemic and related global travel restrictions [10,11,12]. The majority of TALD cases are associated with hotel stays; therefore, the ability of hotels to provide adequate infection control can be decisive in limiting the incidence of TALD.

In Latvia, domestic and foreign tourism is an important part of the national economy. During the previous years, Latvia had an average of 1.5 cases of LD per 100,000 inhabitants, and several cases of TALD have been reported by persons staying at hotels in Latvia [10]. There are no specific national guidelines in Latvia with regard to the prevention and control of Legionnaires’ disease in hotels. Moreover, no data were available on the distribution of *Legionella* in hotel water supply systems; thus, the aim of the current study was to assess the prevalence and genetic diversity of *Legionella* spp. in hot water systems of hotels in Latvia.

## 2. Materials and Methods

### 2.1. Sampling

In total, 834 hot water samples (one liter each) were collected in sterile bottles from the water systems of 80 hotels in Latvia. Samples were collected from November 2016 until June 2022 at 54 hotels in Riga, the capital city of Latvia with high intensity of tourism and more than 600,000 inhabitants and at 26 hotels located in 15 regional towns in Latvia with less than 80,000 inhabitants and low intensity of tourism.

At each hotel, the samples were taken from at least 3 places, such as faucets and shower heads in hotel rooms, heating units, gyms, locker rooms, and SPA facilities. The samples were taken without prior water flushing and after 3 min of flushing. The sampling points and the total number of samples at each hotel were selected depending on the size of the hotel, the accessibility of facilities on the day of sampling, and the amenities available at each hotel. The water temperature was measured during sampling. A specially designed and equipped vehicle was used to transport the samples to ensure a temperature of 0–6 °C during transportation. Testing of the samples was started no later than 6 h after the sampling.

### 2.2. Culturing of Legionella

The isolation and identification of *Legionella* spp. were carried out according to ISO 11731 standards [13]. Each water sample (1 L) was filtered and concentrated using a 0.45 μm polyamide membrane filter (Millipore, Molsheim, France). The filter membranes were resuspended in sterile distilled water (5 mL), shaken for two minutes (Vortex Genius, IKA, Staufen, Germany), and kept at room temperature for 10 min. A total of three 0.1 mL aliquots (untreated, heat treated, and acid treated) were spread on Buffered charcoal yeast extract agar (BCYE, OXOID, Basingstoke, UK) and Glycine vancomycin polymyxin B cycloheximide agar (GVPC, Oxoid, Basingstoke, UK). For samples taken before November 2017, only GVPC medium was used.

The plates were incubated at 36 °C in a humidified environment for 10 days and examined every day starting from Day 3. At least three characteristic colonies from each GVPC plate were selected for subculture on Buffered Charcoal Extract agar medium (BCYE, OXOID, Basingstoke, UK) and Buffered Charcoal Extract agar medium without L-cysteine (BCYE-Cys, OXOID, Basingstoke, UK), and incubated for at least 48 h at 36 °C.

Suspected *Legionella* colonies were identified by matrix-assisted laser desorption/ionization time of flight mass spectrometry (MALDI-TOF MS, Bruker, Bremen, Germany). An agglutination test (Thermo Fisher Scientific, Bred, Netherlands) was used for the confirmation of *L. pneumophila.* Individual latex reagents (Pro-Lab Diagnostics, Richmond Hill, Canada) were used for the exact detection of each *L. pneumophila* serogroup. Colonies from all plates were counted and confirmed, and the estimated number of *Legionella* was expressed as CFU/liter of *Legionella* species and serogroup. All confirmed *Legionella* isolates were obtained in pure cultures and transferred to the culture collection for long-term storage.

### 2.3. DNA Extraction 

From all *L. pneumophila*-positive samples, 79 isolates from 24 hotels were selected for molecular typing. All isolates were deposited in the culture collection and stored at −80 °C. The isolates were thawed and cultured on Buffered Charcoal Yeast Extract (BCYE, Oxoid, Basingstoke, UK) agar at 37 °C for 48 h before typing. DNA extraction was performed after 48 h incubation of *L. pneumophila* at 36 ± 1 °C. Single colonies were suspended in tubes with 500 µL nuclease-free water and then homogenized with a vortex to obtain homogenous suspensions. Lysis was carried out by thermal shock using 8 min of incubation at 100 °C temperature. The tubes were cooled and centrifuged (3 min × 13,000 rpm) to obtain a supernatant, and approximately 400 μL of supernatant was transferred to new tubes. DNA concentration was determined with a spectrophotometer (NanoDrop Technologies, Waltham, MA, USA).

### 2.4. Sequence-Based Typing

Sequence-based typing (SBT) was conducted in accordance with the European Society of Clinical Microbiology and Infectious Diseases (ESCMID) Study Group for *Legionella* Infections (ESGLI) sequence-based typing (SBT) scheme [14]. Briefly, PCR amplification with *Taq* DNA polymerase (Qiagen, Hilden, Germany) was performed for extracted DNA, then PCR products were analyzed by capillary gel electrophoresis on a QIAxcel Advanced instrument (Qiagen, Hilden, Germany). PCR products were purified and prepared for the sequencing reaction. Sanger sequencing reaction was performed using BigDye Terminator v3.1 Cycle Sequencing Kit protocol and read with Genetic Analyzer 3500 (Applied Biosystems, Waltham, MA, USA). New allelic profiles were submitted to the ESGLI SBT database [15].

### 2.5. Whole Genome Sequencing

All 79 isolates were subjected to whole-genome sequencing. DNA for library preparation was extracted from single colonies using the QIAamp DNA Mini kit (Qiagen, Hilden, Germany) following the manufacturer’s instructions. DNA libraries were prepared with the Illumina DNA Prep DNA library preparation kit (Illumina, San Diego, CA, USA). Sequencing was performed using Illumina MiSeq Reagent Kit v2 with 500 cycles or v3 with 600 cycles (Cat# MS-102-2003 and MS-102-3003) to obtain paired-end reads in at least 30× coverage. Sequencing adapters and low-quality bases were trimmed from the reads using Trimmomatic v0.38 [16]. De novo assembly of the trimmed reads was performed by the SPAdes assembler v3.14.0 [17] in “--isolate” mode. 

SBT, according to the ESCMID *Legionella* Study Group (ESGLI) scheme [14,18], was performed on the assembled genomes and raw reads using a combination of two software tools. First, the legsta tool (Seemann, https://github.com/tseemann/legsta/ accessed on 15 November 2022) was employed to identify alleles of each SBT locus in the assembled genomes. Since multiple different copies of the *mompS* gene can be present in one genome, a specialized *mompS* tool [19] was applied to extract the consensus sequence of the correct *mompS* locus from raw reads and to determine its allele. The SBT allelic profiles returned by both tools were compared, and the *mompS* allele reported by the second tool was assumed to be the correct one when there was a discrepancy between the outputs of both tools.

Core genome multilocus sequence typing (cgMLST) genotypes were determined according to the *L. pneumophila* cgMLST scheme developed by Moran-Gilad et al. [20]. The cgMLST scheme, consisting of 1521 loci, was preprocessed and adapted to be used for allele calling by chewBBACA software v2.8.5 [21]. This tool called alleles in assembled genomes and identified two loci as possibly paralogous; therefore, only 1519 loci were considered in the cgMLST analysis. 

### 2.6. Data Analysis

R version 4.2.1 (23.06.2022. ucrt), 2022 (The R Foundation for Statistical Computing, Vienna, Austria) was used for data analysis. Map was designed using QGIS 3.26.1. Genotypes were visualized in the form of minimum spanning trees with GrapeTree v1.5.0 [22]. 

## 3. Results

### 3.1. Prevalence of Legionella

In total, 834 hot water samples from 80 hotels were tested (Table 1). Overall, in 14 out of 15 regional towns, *Legionella*-positive samples were detected. Sigulda was the only regional town of 15 where no positive samples were found.

At least one *Legionella* spp.-positive sample was detected in 47 out of 80 hotels (58.8%). In Riga, where hotels are supplied with treated surface water, the proportion of *Legionella* spp.-positive hotels was lower than in other cities and towns of Latvia receiving water from underground aquifers, but this difference was not statistically significant (*p* = 0.12). Overall, 235 out of 834 samples (28.2%) were *Legionella* spp. positive; however, the proportion of positive samples was significantly higher in the hotels outside Riga (*p* < 0.0001).

In the hotels where at least one *Legionella* spp. positive sample was detected, the most prevalent *Legionella* species was *L. pneumophila*, which was found in 46 out of 47 (98%) cases. In 43 (91%) hotels, *L. pneumophila* was the only species found during the period of study, while *L. pneumophila* and *L. rubrilucens* were found in two hotels. In one hotel, *L. pneumophila* and *L. anisa* were detected, and in one hotel *L. rubrilucens* was the only species.

Different levels of colonization by *L. pneumophila* were observed during the study. The concentration of *L. pneumophila* varied from 50 CFU/L in 11 samples from 11 hotels up to 1.1 × 10^4^ CFU/L in two samples from two hotels (Table 2). In total, *L. pneumophila* exceeded 1 × 10^3^ CFU/L in 76 out of 230 (33%) positive samples, and at least one *L. pneumophila* positive sample with level of colonization over 1 × 10^3^ CFU/L was found in 26 out of 47 (55%) hotels. It was observed that hotels located outside Riga supplied from underground aquifers showed a significantly higher average level of *L. pneumophila* (*p* = 0.02).

### 3.2. Temperature of Hot Water

The temperature of hot water before and after flushing was measured during the sampling (Table 3). Data analysis showed that the temperature of hot water in 28 out of 80 hotels (35%) did not reach 50 °C after flushing, while the temperature of hot water was between 50 °C and 55 °C in 22 hotels (27%) and exceeded 55 °C in 30 hotels (38%) at the point of water use. The increase in hot water temperature after three minutes of flushing varied from 8.4 °C to 39.0 °C. On average, the temperature after flushing increased by 23.8 ± 1.2 °C.

The average water temperature in hotels was not significantly different between Riga and other cities or towns neither before (*p* = 0.97) nor after (*p* = 0.66) the flushing. Although the water temperature in the majority of hotels did not exceed 55 °C, it was observed that flushing could significantly increase the water temperature at the point of use (*p* < 0.0001). Data analysis did not reveal any significant relationships between the temperature of the hot water and the level of *L. pneumophila* colonization.

### 3.3. Levels of Colonization

The analysis of data on the levels of *L. pneumophila* colonization (Table 4) revealed a statistically insignificant decrease in *L. pneumophila* after flushing (*p* = 0.16). However, our data also showed that in 53% of hotels where the level of *L. pneumophila* exceeded 1.0 × 10^3^ CFU/L (4.4 × 10^3^ CFU/L on average), the flushing of water reduced the colonization levels below 1.0 × 10^3^ CFU/L (3.0 × 10^2^ CFU/L on average) at the point of water use. 

### 3.4. Serotyping

Serotyping data analysis revealed that the most common *L. pneumophila* serogroup was SG3, which was found in 113 (49.8%) positive samples from 27 hotels. SG2 was detected in 69 (30.4%) positive samples from 20 hotels, SG1 in 31 (13.7%) samples from 11 hotels, SG9 in 6 (2.6%) samples from 2 hotels, and SG6 (2.2%) in 5 samples from 3 hotels. In two samples (0.9%) from one hotel, both SG2 and SG3 were detected.

### 3.5. Sequence-Based Typing

**Figure 1:**
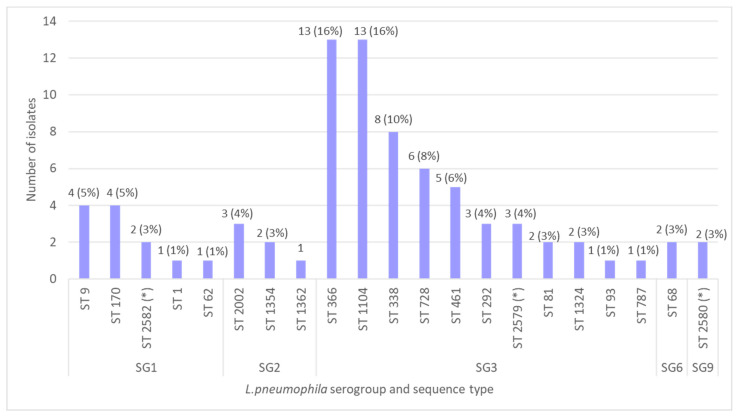
The distribution of *L. pneumophila* serogroups and sequence types in water samples from hotels (*—new sequence types).

Among all *L. pneumophila*-positive samples, 79 isolates from 24 hotels were selected for genetic analysis that was carried out using SBT. As a result, 21 different sequence types were obtained, including three new types: ST2582, ST2579, and ST2580 (Figure 1). The most prevalent sequence types were ST1104 (16%), ST366 (16%), ST338 (10%), and ST728 (8%).

**Figure 2:**
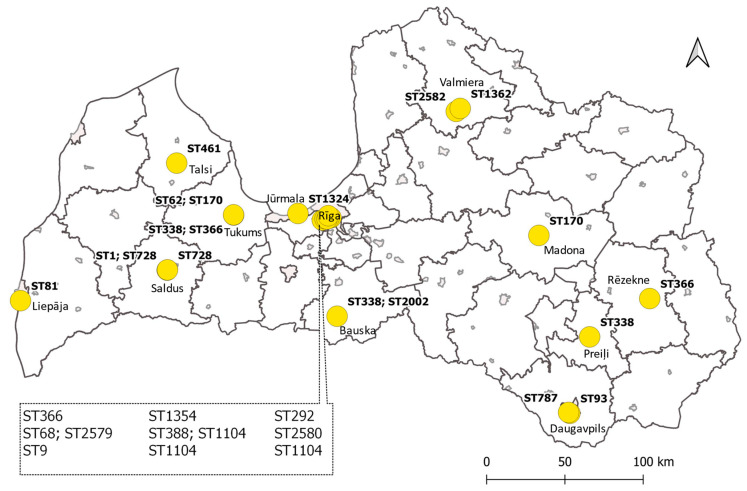
Geographical distribution of *L. pneumophila* sequence types in Latvia.

In hotels supplied with treated surface water, nine sequence types—ST9, ST68, ST292, ST338, ST366, ST1104, ST1354, ST2579, and ST2580—were detected in 36 water samples, while in areas receiving water from underground aquifers, 14 sequence types were detected in 43 samples (Figure 2). Only three sequence types—ST170, ST338, and ST366—were found both in hotels supplied with surface and underground water.

Only one *L. pneumophila* sequence type was detected in 18 out of 24 (75%) hotels, while two different sequence types were found in six (25%) hotels during the study period. Among the six hotels with two *L. pneumophila* sequence types, four were located outside Riga.

**Figure 3:**
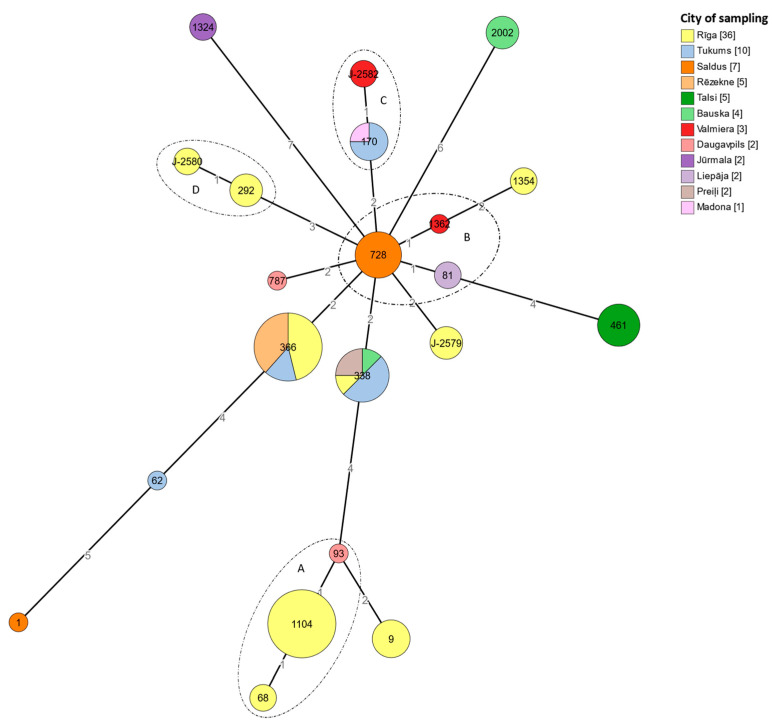
A minimum spanning tree of 79 *L. pneumophila* isolates from water samples taken in hotels in Latvia, based on SBT. The node sizes are proportional to the numbers of isolates sharing an identical pattern. Each node color represents the geographic origin of the isolate. The relative branch length scaling is maintained to project the internodal distance. The clonal complexes (A–D) generated at a single locus variant level are indicated by the circles surrounding the nodes.

A minimum spanning tree was constructed with 21 sequence types with at least six matching alleles, as well as four clonal complexes (Figure 3). All four identified clonal complexes included isolates belonging to different serogroups. The clonal complexes were formed regardless of the sampling geography. Only the clonal complex D was found in two isolates from the same city, while the other clonal complexes included isolates from different cities and towns.

### 3.6. cgMLST Typing

**Figure 4:**
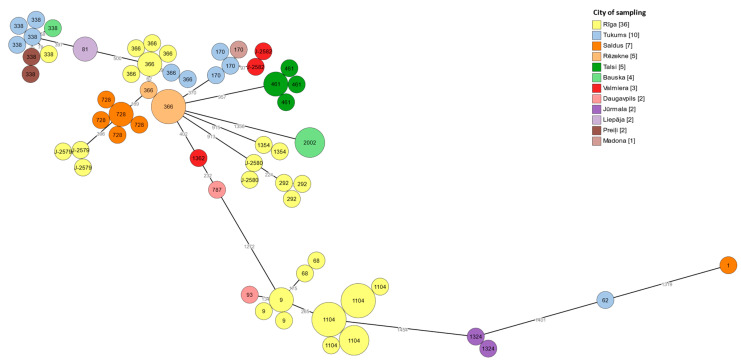
A minimum spanning tree of 79 *L. pneumophila* isolates from water samples taken in hotels in Latvia, based on cgMLST. Numbers on nodes correspond to SBT sequence types. The node sizes are proportional to the numbers of isolates sharing an identical pattern. Branches between the nodes show the genetic distance between them. Each node color represents the geographic origin of the isolate.

A total of 61 genotypes were obtained by using cgMLST typing (Figure 4). No sequence types that were unique to one city or region were identified. Sequence types found in one region may also be found in other regions, and *L. pneumophila* strains found in one city or town may belong to different clonal complexes. Thus, ST461 detected only in five different samples from the same building in the town of Talsi, appears as one node in the SBT minimum spanning tree but as four separate cgMLST types with minor differences in cgMLST, where two isolates were identified as identical and three isolates as different.

## 4. Discussion

Hotels, as the main element of the hospitality industry, can play an important role not only in promoting tourism but also in safeguarding public health. However, Latvia has been associated with TALD cases in the annual reports of the European Centre for Disease Prevention and Control. In this study, we analyzed the prevalence of *Legionella* in the water supply systems of 80 hotels in Latvia. Overall, 28% of the samples were positive for *Legionella,* and at least one *Legionella*-positive sample was found in 58.8% of the hotels (47 out of 80). Furthermore, *Legionella* concentration exceeded 1000 CFU/L in 33% of the cases, which may be recognized as a high level of contamination that may endanger the health of hotel guests.

A similar occurrence of *Legionella* has been reported from earlier studies: 20.7% of samples from 62.95% of sampled hotels in Greece [23], 25.6% of samples from 57.15% of sampled hotels in Italy [24], 15.9% of hot water samples from 65.4% of sampled facilities of the Balearic Islands, Spain [25], and 17% samples from 60% of sampled hotels in Israel [26]. *Legionella* was also detected in 25.7% of samples from hotels in Bosnia and Herzegovina [27]. The lowest prevalence of *Legionella* was observed in the Canary Islands—only 8.5% of samples from hot water distribution systems were found to be contaminated [28]. Generally, with the exception of the study by Domenech-Sanchez [28], a fairly similar prevalence of *Legionella* has been detected. However, it is quite difficult to unambiguously compare the data, as there were significant differences in the sampling plans, which covered large monitoring programs, convenience sampling, and targeted sampling programs in response to outbreaks of Legionnaires’ disease. Different countries may have different monitoring and control requirements and the required minimum limits for hot water temperature.

The average hot water temperature in Latvian hotels was 49.8 °C, and in 62% of hotels, the hot water temperature did not exceed 55 °C after flushing. The temperature of 55 °C was identified as a cutoff point, above which there was a strong negative trend in *Legionella* colonization [29].

In our study, the samples were collected regardless of previous cases of Legionnaires’ disease. In addition to that, Latvia is located in a temperate climate zone, unlike most countries featured in previous studies, were Italy, Spain, and Israel are located in the subtropical climate zone. While climatic conditions can increase the risk of Legionnaires’ disease [30], the observed prevalence of *Legionella* in Latvian hotels was similar to the prevalence of *Legionella* in more southern countries, which may indicate that the lack of clear requirements regarding water temperature allows *Legionella* to proliferate in the water supply systems of hotels.

Remarkably, the proportion of positive samples was significantly higher in hotels outside Riga (48%), while only 23% of samples from Riga hotels tested positive. In the city of Riga, treated surface water is used for water supply, while underground water in the regions is not additionally treated. The incoming municipal water in Riga is thus better disinfected. However, in our opinion, the main reason for lower *Legionella* occurrence in Riga may be better management practices, and quality standards since most of the hotels in the capital city belong to international hotel chains. On the other hand, regional hotels are most often smaller and less occupied, which can result in frequent water stagnation associated with the proliferation of *Legionella*. 

Among all Legionella-positive hotels, *L.pneumophila* was found in 46 hotels out of 47 (98%). It was the sole *Legionella* species in 91% of hotels, while in three cases, it was found in co-culture with *L.anisa* and *L.rubrilucens*, similar to previous studies [23,25]. However, the classic culture method [13] has not been designed to find and identify all species in a sample, and it should be considered that non-pneumophila *Legionella* is often underdetected.

In our study, 49.8% of *L. pneumophila* isolates belonged to SG3, while SG1, which is considered the most common cause of Legionnaires’ disease, was detected in only 13.7% of cases. However, it should be noted that the first-choice test is still the urine antigen test, which is specific for *L. pneumophila* SG1, and only 11% of Legionnaires’ disease cases in Europe are culture-confirmed [10]; therefore, it is possible that cases of Legionnaires’ disease associated with other serogroups remain underdiagnosed. Meanwhile, in Latvia until 2022, the identification and confirmation of clinical cases of Legionnaires’ disease were mostly carried out using a urine antigen test and diagnostic methods not related to cultivation; accordingly, no clinical isolates have been obtained yet, and data on the characteristics of clinical isolates in Latvia are not available. Therefore, we consider that the number of Legionnaires’ disease cases in Latvia is underdiagnosed and underreported.

Our study represents SBT analysis of 79 environmental *L. pneumophila* isolates obtained from municipal water at Latvian hotels, resulting in the identification of 21 different sequence types (ST), including three new STs. Previous studies have also described a high diversity of STs; for example, 27 different STs were identified in 78 isolates from Israel [26], 88 *L. pneumophila* isolates were divided into 33 STs in a study from Slovenia [31], while researchers from Canada reported that 141 sporadic cases of Legionnaires’ disease belonged to 57 different STs [32]. All these studies also identified new STs. The identification of new STs suggests that the genetic composition of *Legionella* strains may be unique in the region and may differ significantly from described human and environmental isolates in other countries [33]. Recombination and gene transfer between different *Legionella* species and strains are the main reason for the high genetic diversity [34,35].

We found that the most widely represented sequence types were ST338, ST366, and ST1104. At least 15 more STs found in Latvia have been mentioned in other studies as clinical isolates from sporadic cases, outbreaks, and travel-related cases in different countries of the world [31,32,36,37,38,39]. These findings suggest that *L. pneumophila* strains persisting in water supply systems in Latvia may pose a public health risk under certain conditions.

ST1 is the most widely represented *Legionella* ST in the world; however, this ST was identified only once during our study. Based on research from other countries, this can be explained by the locally limited expansion of ST types in the region [38]. For example, the dominant ST in several European countries—Belgium [36], United Kingdom [40], France [41], and the Netherlands [42]—is ST47, which has not been found in Latvia. However, it should be noted that there are little data on Eastern Europe, and the geographically closest studies come from Poland, where ST47 has not been mentioned either [37], providing support for the importance of local genetic variants.

We were able to compare classical SBT typing with WGS-based SBT typing and cgMLST typing. In addition to the convenience of WGS-based typing, we confirmed the practical value of this method. Using visualization of cgMLST genotypes (Figure 4), we observed that compared to SBT (Figure 3), isolates that appear as one node with SBT can be classified as different isolates in cgMLST, such as ST 728, ST1104, and ST461, supporting the theory that the cgMLST typing method analyzing 1519 loci provides much better resolution in the evaluation of *L. pneumophila* than the SBT scheme based on the analysis of allelic profiles of only seven loci [20]. A better resolution of the method can be particularly important in epidemiological investigations when the relationship between a case and a possible source of infection needs to be confirmed [43,44].

During this study, high *Legionella* contamination and a considerable genetic diversity were found in hotel water supply systems in Latvia, which can be explained by the lack of regulatory requirements and certain control measures with regard to the temperature of hot water. Our results confirmed that Latvian hotels can be a source of TALD, and additional preventive measures are needed to ensure comprehensive control of *Legionella*. In the meantime, continuous monitoring with integrated studies to characterize and compare clinical and environmental isolates is needed to better understand the hotspots and persistence of *Legionella* in water supply ecosystems and their ability to infect humans.

## Figures and Tables

**Table 1:** Prevalence of *Legionella* spp. in water samples from hotels in Latvia.

Location of Hotel	Total Tested		*Legionella* spp. Positive	
	No. of Hotels (%)	No. of Samples (%)	No. of Hotels (%)	No. of Samples (%)
Riga	54 (67.5)	683 (81.9)	28 (51.9)	162 (23.7)
Other cities and towns	26 (32.5)	151 (18.1)	19 (73.1)	73 (48.3)
Total	80 (100)	834 (100)	47 (58.8)	235 (28.2)

**Table 2:** *L. pneumophila* colonization level (CFU/L) in hotels supplied from different water sources.

	Water Source in Hotels		Total
Level of Colonization, CFU/L	Surface Water	Underground Aquifers	
Min	50	50	50
Max	1.1 × 10^4^	1.1 × 10^4^	1.1 × 10^4^
Average	1.0 × 10^3^ ± 1.3 × 10^2^	1.6 × 10^3^ ± 2.6 × 10^2^	1.2 × 10^3^ ± 1.2 × 10^2^

**Table 3:** The hot water temperature before and after flushing.

Temperature, °C	Before Flushing	After Flushing
Min	16.2	27.7
Max	62.9	68.8
Average	35.7 ± 0.7	49.8 ± 0.4
Mode	27.0	47.0

**Table 4:** Level of *L. pneumophila* colonization before and after flushing.

Level of Colonization, CFU/L	Before Flushing	After Flushing
Min	50	50
Max	1.1 × 10^4^	9.0 × 10^3^
Average	1.7 × 10^3^ ± 2.8 × 10^2^	1.2 × 10^3^ ± 1.8 × 10^2^

## Data Availability

Raw sequence reads have been deposited at the European Nucleotide Archive under the project accession number PRJEB59332.
